# Human Myocardial Protein Pattern Reveals Cardiac Diseases

**DOI:** 10.1155/2012/342659

**Published:** 2012-08-08

**Authors:** Jonas Bergquist, Gökhan Baykut, Maria Bergquist, Matthias Witt, Franz-Josef Mayer, Doan Baykut

**Affiliations:** ^1^Analytical Chemistry, Department of Chemistry, Biomedical Center and SciLife Lab, Uppsala University, P.O. Box 599, 751 24 Uppsala, Sweden; ^2^Bruker Daltonik GmbH, 28359 Bremen, Germany; ^3^Department of Medical Sciences, Hedenstierna Laboratory, Uppsala University, 75185 Uppsala, Sweden; ^4^Institute of Biophysics, University of Frankfurt, 60438 Frankfurt/M, Germany

## Abstract

Proteomic profiles of myocardial tissue in two different etiologies of heart failure were investigated using high performance liquid chromatography (HPLC)/Fourier transform ion cyclotron resonance mass spectrometry (FT-ICR MS). Right atrial appendages from 10 patients with hemodynamically significant isolated aortic valve disease and from 10 patients with isolated symptomatic coronary heart disease were collected during elective cardiac surgery. As presented in an earlier study by our group (Baykut et al., 2006), both disease forms showed clearly different pattern distribution characteristics. Interesting enough, the classification patterns could be used for correctly sorting unknown test samples in their correct categories. However, in order to fully exploit and also validate these findings there is a definite need for unambiguous identification of the differences between different etiologies at molecular level. In this study, samples representative for the aortic valve disease and coronary heart disease were prepared, tryptically digested, and analyzed using an FT-ICR MS that allowed collision-induced dissociation (CID) of selected classifier masses. By using the fragment spectra, proteins were identified by database searches. For comparison and further validation, classifier masses were also fragmented and analyzed using HPLC-/Matrix-assisted laser desorption ionization (MALDI) time-of-flight/time-of-flight (TOF/TOF) mass spectrometry. Desmin and lumican precursor were examples of proteins found in aortic samples at higher abundances than in coronary samples. Similarly, adenylate kinase isoenzyme was found in coronary samples at a higher abundance. The described methodology could also be feasible in search for specific biomarkers in plasma or serum for diagnostic purposes.

## 1. Introduction

Understanding the differences of proteomic profiles has a crucial importance for gaining insight into molecular mechanisms of disease. Although the molecular origin of the cardiac dysfunction is still largely unknown in the majority of heart diseases [1, 2], heart insufficiency is increasingly expected to be a result from alterations in gene and protein expression. Gene expression is a more static process; however, protein patterns may be altered faster in close relationship with the appearance of disease, making up an adequate identification of proteome characteristics important. If different etiologies and pathways of cardiac disease progression can be represented in the form of unequivocal proteomic patterns, these patterns may help simplify and accelerate the diagnostics and be used as an appropriate diagnostic method in daily clinical routine [3–6]. Molecular differences extracted out of these patterns may also reveal potential biomarkers that could be targeted for screening purposes [7, 8]. A possibility to locate some of these molecules in circulating blood, where an easy access for high-throughput assays would be valid, is especially attractive.

The objective of this study is to evaluate selected individual myocardial samples which are representative for aortic valve disease (AVD) and coronary heart disease (CHD), respectively. As presented in our recent study [9], proteomic pattern distribution characteristics of myocardial tissue in AVD and CHD were found to be clearly different using liquid-chromatography Fourier transform ion cyclotron resonance mass spectrometric (LC-FT-ICR MS) analysis. Classification patterns obtained from the comparison of LC-FT-ICR MS of digested proteins in the samples could be used for correctly sorting them in exact categories. The endogenous proteins were analyzed using a “bottom-up” proteomic approach that starts by a tryptic digestion of all the proteins in the samples and then analyzing the digests through LC and online electrospray ionization of the analytes into a quadrupole/hexapole FT-ICR MS. After isolation in the quadrupole, collision-induced dissociation (CID) of selected peptides can be performed in the hexapole collision cell, and using the acquired fragment spectra, proteins can be identified by database searches. For comparison and further validation, selected peptides were also fragmented and analyzed using liquid-chromatography-matrix-assisted laser desorption-ionization time-of-flight/time-of-flight mass spectrometry (LC-MALDI-TOF/TOF MS). With the combination of these techniques, the measurement of peptides from LC-separated samples results in precise mass information on the ppm-level, in very high resolution and in high sensitivity that in itself has been successfully used to explore the protein content of body fluids, for example, plasma and cerebrospinal fluid (CSF) [10–13]. In addition, sequence information essential for significant protein identification in minute tissue samples, for example, laser-dissected human spinal cord tissue can be obtained [14].

## 2. Materials and Methods

**Figure 1:**
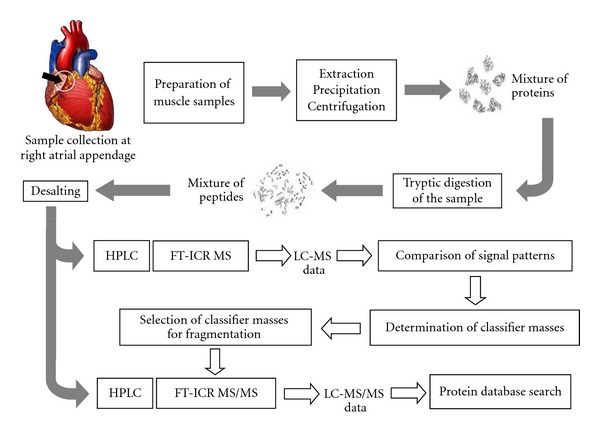
Flowchart for the path of the sample preparation and measurements with 9.4 Tesla FT-ICR MS. The measurement of the samples with two different instrument had the reason that the samples were measured with a classical FT-ICR instrument without external MS/MS capability. After the differential mass spectrometric runs followed by the pattern comparison, a quadrupole/hexapole FT-ICR instrument with external MS/MS capability was available. The fragmentation studies for the protein identification were performed with this latter instrument.

As a general overview for the method used, a flow chart is shown in Figure 1 depicting the sample preparation, mass spectrometric methods and measurements, as well as the data evaluation.

### 2.1. Sample Selection and Data

In 20 patients undergoing cardiac surgery with extracorporeal circulation, right atrial appendages were subject to removal and discarding for venous cannulation that were collected intraoperatively, after approval by the local ethical committee. The group of patients consisted of 10 individuals with hemodynamically significant, isolated AVD and 10 with isolated symptomatic CHD. Patient selection was made in such a way that all patients with CHD selected for this study were “healthy” in terms of the AVD and all selected AVD patients were “healthy” in terms of the coronary disease. The median age was 62 years (45–81) in the AVD group, and 66 years (37–83) in the CHD group. All antithrombotic agents were suspended 10–14 days prior to surgery. In all studied cases, patients stopped taking aspirin one week before the operation. Cardiac samples (each of them in the range of 1 cm^3^) were immediately washed in Krebs-Henseleit solution (118 mM NaCl, 4.7 mM KCl, 1.2 mM MgSO_4_, 1.25 mM CaCl_2_, 1.2 mM KH_2_PO_4_, 25 mM NaHCO_3_, 11 mM glucose) and fixed on wax plates at room temperature. After separation from the epicardium, the trabecular tissue was shock-frozen in liquid nitrogen and stored at −75°C.

### 2.2. Sample Preparation

Sample selection and preparation of the tissue specimens have been described in detail in our earlier study [9]. Briefly, the tissues were homogenized in 8 M urea, 0.4 M NH_4_HCO_3_ followed by reduction of the disulfide bridges, carbamidomethylation of cysteins, and tryptic digestion according to the manufacture's protocol (Modified Trypsin, sequencing grade, Promega GmbH). The digests were SPE extracted (Spec Plus C_18_ AR, Ansys Diagnostics Inc., CA), vacuum-centrifuged to dryness, and then redissolved in 0.1% formic acid before injection into an LC system. Acetonitrile and acetic acid, formic acid and triflouro acetic acid were purchased from Merck (Darmstadt, Germany). Urea, ammonium carbonate, and iodoacetamide were obtained from Sigma Chemical Co. (St. Louis, MO, USA) and were used without further purification. Dithiothreitol was purchased from Amersham Biosciences (Uppsala, Sweden). All used chemicals were of analytical grade. Trypsin, sequence grade from bovine pancreas (1418475), was obtained from Roche Diagnostics (Mannheim, Germany). Water was purified with a Milli-Q purification system (Millipore, Bedford, MA, USA). All fused-silica capillaries were obtained from Polymicro Technologies (Phoenix, AZ, USA).

### 2.3. LC/FT-ICR MS/MS for Protein Identification

The total amount of each digested sample was dissolved in 250 *μ*L 0.1% trifluoroacetic acid (TFA), split into 5 vials each of 50 *μ*L sample solution and Speed Vacced to dryness at medium temperature. The dried sample of one vial of each sample was dissolved in 50 *μ*L 0.1% TFA and diluted 1 : 10 with 0.1% formic acid. An aliquot of 5 *μ*L of the sample solution was injected on a Nano LC column (see below) for FT-ICR-NanoLC/MS/MS analysis.

**Figure 2:**
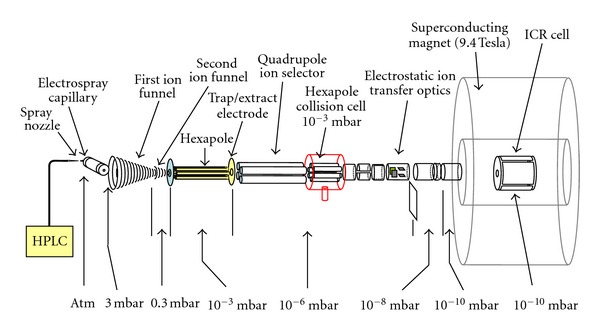
Non-scaled schematic view of an LC-FT-ICR MS system with a quadrupole mass selector and a hexapole collision cell. In the electrospray ion source the formed ions are captured after they pass the electrospray capillary in an ion funnel, which increases the sensitivity of the system by roughly up to an order of magnitude. Ions are transferred through two ion funnels into a hexapole ion guide, where they can also be trapped. Ions are selected in the quadrupole mass selector and can undergo collision induced dissociation in the hexapole collision chamber which is at a relatively high pressure. The ICR cell is in the magnetic center of a 9.4T superconducting magnet. The vacuum system, not shown in the figure, consists of pumping stages down to the range of 10^−10^ mbar in the ultra high vacuum chamber of the ICR cell. The numbers shown are approximate pressures in different pumping stages.

Mass spectra were acquired with an APEX Qe FT-ICR (Bruker Daltonics Inc., Billerica, MA, USA) equipped with a 9.4 Tesla superconducting magnet (Bruker BioSpin, Wissembourg, France). A schematic description of the LC-FT-ICR MS system with a quadrupole mass selector and a hexapole collision cell is shown in Figure 2. Samples were ionized in an “Apollo II” electrospray ion source (Bruker Daltonics, Billerica, MA, USA) with ion funnel technology for efficient capture of the generated ions. Ions were detected in a cylindrical ion cyclotron resonance cell with segmented trapping plates (infinity cell) [15]. Nano LC-MS/MS measurements were performed with a Nano LC system Ultimate 3000 (Dionex, Sunnyvale, CA, US) fitted with 2 10-port valves. An online-nanospray ESI source (Bruker Daltonics, Billerica, MA, USA) was coupled to the NanoLC system. This ion source was equipped with an angled off-axis spraying system, that used a PicoTip adapter (New Objective, MA, USA) to connect the tubing of the NanoLC column to a distal-coated fused silica needle that had a 10 *μ*m inner diameter (New Objective, MA, USA). A spring in the needle holder provided the connection from the needle to ground potential. The tip of the needle was placed a few mm in front of the orifice of the glass capillary at an angle of approximately 70°. The voltage applied to the metal-coated capillary entrance of the electrospray source was set to −1500 V. The NanoLC column PepMap C-18 (75 *μ*m inner diameter, 15 cm length, 5 *μ*m particle size, Dionex, Sunnyvale, CA, USA) was used for compound separation with a flow of 200 nL/min. The peptides were first captured on a C-18 precolumn for desalting of the sample and eluted from this column to the PepMap column with a 185 min gradient for peptide separation of highly complex sample (solvent A: water with 0.1% formic acid, solvent B: acetonitrile with 0.1% formic acid, 0 min 2% solvent B, 5 min 2% solvent B, 175 min 40% solvent B, 190 min 50% solvent B, 191 min 90% solvent B, 205 min 90% solvent B, 206 min 2% solvent B, 250 min 2% solvent B). After storing the sample on the precolumn, this column was washed for 5 minutes with 0.1% TFA for desalting using a 30 *μ*L/min flow and than switched to gradient flow. The pressure in the collision cell (located between the quadrupole filter and the ICR analyzer cell) for CID was set by increasing the read-out pressure of the source ion-gauge to about 4.5 × 10^−6^ mbar (approx., a factor of 10 higher than without pressure in the collision cell). The default parameter setting was used for collision energy calculation for CID fragmentation. The FT-ICR mass spectra were acquired with the acquisition software Apex Control 1.0 (Bruker Daltonics, Billerica, MA, USA) using data-dependent MS/MS acquisition. The ion accumulation time was set to 0.5 s for the MS scan and 2 s for the MS/MS. The mass range was set to m/z 246 to m/z 2000 for the MS and MS/MS spectra using 512 k data points. The mass spectra were processed with 512 k data points and sine apodization. Further processing of the data including mass deconvolution and mgf-file generation for database search was performed with Data Analysis 3.3 (Bruker Daltonics, Billerica, MA, USA).

The mass spectra were processed using the protein analysis software Biotools 3.0 (Bruker Daltonics, Billerica, MA, USA) for database searches and results interpretation. The MASCOT search engine (Matrix Science, London, UK) [16, 17] was used to search the SwissProt database. MOWSE (molecular weight search) scores, assigned by MASCOT, were calculated based on the algorithm described by Pappin et al. [18]. Search criteria for all experiments included one missed cleavage, taxonomy human, fixed modification carbamidomethyl, and variable modifications oxidation of methionine. Mass tolerances for the MS/MS search were set to 4 ppm for the parent mass and 0.01 Da for the fragment masses. In a second search, 2 missed cleavages were allowed, and in a third search, also unspecific cleavages were accepted. Mass spectra were externally calibrated with a siloxane mixture of the chemical background signals using a two parameter linear calibration [19].

### 2.4. LC/MALDI TOF/TOF MS for Protein Identification

*α*-cyano-hydroxy cinnamic acid (CHCA) and the profiling kit Magnetic Beads based on Weak Cation Exchange Chromatography (MB-WCX) were obtained from Bruker Daltonik, Bremen, Germany. HPLC gradient grade and ammonium citrate, dibasic, ACS grade, were obtained from Sigma-Aldrich (Steinheim, Germany).

The capillary high-performance liquid chromatography (HPLC) separations were performed using an Agilent 1100 Cap-LC system equipped with a 15 cm × 180 *μ*m I.D. PepMap column with a 3 *μ*m C_18_ stationary phase (LC Packings, Amsterdam, The Netherlands). For all separations eluent A consisted of 0.1% TFA/water and 2% CH_3_CN and eluent B was 0.05% TFA in 100% CH_3_CN. An isocratic flow of eluent A, 2 *μ*L/min for 5 min, was followed by a gradient from 2% to 40% CH_3_CN in 90 minutes. All separations were performed at ambient temperature and the injection volume was 8 *μ*L. After each 15 s fractions of 0.5 *μ*L were dispensed onto a spot on the MALDI target.

**Figure 3:**
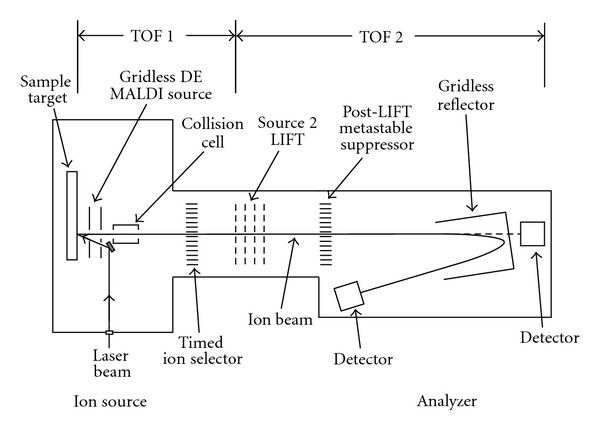
Non-scaled schematic view of a MALDI-TOF/TOF mass spectrometer.

The MALDI-TOF-MS instrument used was an Ultraflex II TOF/TOF (Bruker Daltonik GmbH, Bremen, Germany) [20]. It was equipped with a Smart Beam laser [21]. The focus diameter was approximately 60 *μ*m. All spectra were acquired using the reflectron mode of the instrument (Figure 3) at 25 kV acceleration voltage. While the MS data was recorded in a fully automated fashion the MS/MS data of peptides was acquired in a manual operation mode of the instrument. In order to acquire MS/MS spectra, precursor ions were accelerated out of ion source (S1) by means of time delayed extraction. Fragmentation was performed either by laser-induced or collision-induced dissociation or a combination of both. The collision cell was located right after S1. Argon was used as a collision gas. In the first field free region of the TOF1, the precursor ions including the already created related fragment ions are passed straight through an ion selection unit, while all the rest is deflected. The resolution of this unit is about 750 FWHH (single mass resolution at mass 750 Da). The selected precursor ions and their corresponding fragment ions then enter a second ion source (S2). Here, they are accelerated again, leading to a separation of their mass in the second field free drift region (TOF2). All fragment ions are separated in time and focused in energy according to their *m/z* (z = 1) by a combination of time-delayed extraction out of S2 and time focusing in the reflectron of TOF2. Depending on their mass, typical fragment ion resolution is in the range of 2000–5000.

Despite a 90 min separation of the peptides in the HPLC and a spread of fractions onto 384 spots, the acquired MS spectra were still very complex. On one side, complexity increased suppression effects during desorption/ionization, on the other side, selection of individual peptides for MS/MS often was ambiguous. In order to simplify MS spectra and avoid mixing of fragments from different precursor ions prior to the LC separation, selected samples were prefractionated using Magnetic Beads based on Weak Cation Exchange Chromatography (Profiling Kit 100 MB-WCX, Bruker Daltonik GmbH, Bremen, Germany). The freeze-dried samples were dissolved in 10 *μ*L 0.1% TFA. After a further 1 : 10 dilution, 5 *μ*L was used to bind the peptides to the beads. 8 *μ*L of the supernatant was used in the first run of the LC separation. After washing of the beads with washing buffer, the bound peptides were eluted using 3 × 3 *μ*L of elution buffer. In order to remove the acetonitrile from the eluent, the volume was reduced in a speedvac by a factor of two. The remaining volume of 4 *μ*L was then used for a second run of the LC separation. 

The target of choice for the LC-MALDI approach was a prespotted Anchor Chip target (Bruker Daltonik GmbH, Bremen, Germany). Within a 90 min LC run, all 384 sample spots on the target were prepared. After evaporation of the solvent, the target was washed by dipping it for a few seconds into a solution of 10 mM ammonium citrate/0.1% TFA. The sample plate was then introduced into the Ultraflex II MALDI-TOF mass spectrometer, and mass spectra were recorded from each prepared sample spot. The calibration used was an external near-neighbor calibration by means of additional 96 prespotted calibration spots. The sample used for calibration was a mixture of peptides covering the mass range from 700 Da–3500 Da already prepared onto the disposable target. The acquisition process was controlled by the WARP-LC software (Bruker Daltonik GmbH, Bremen, Germany) and a compound list was created. From these peptide masses, scored according to their signal-to-noise and the complexity of the MS spectrum, the candidates of possible biomarkers were selected manually and their MS/MS spectrum was recorded.

The spectra were processed by means of Flex Analysis and the peak lists were sent to BioTools for the database search using Mascot (Matrix Science, London, UK, [17]). The following search parameters were chosen for the database search. Taxonomy: human; database: SwissProt, variable modification: carbamidomethylation (C) and oxidation (M): MS/MS tolerance: 0.5 Da, Partials: 1. In a second search, 2 missed cleavages were allowed, and in a third search also unspecific cleavages were accepted.

### 2.5. Pattern Recognition and Extraction of Classification Features

The pattern recognition and classification strategy have been described in detail by Ramström et al. [10] and in Baykut et al. [9]. Briefly, a numerical code was developed for the analysis. The first part of the code normalizes the spectrogram intensity, removes the noise, and calibrates the individual HPLC/FT-ICR mass chromatograms in time to a common “table” sample. The second part of the code concerns the calibrated sample classification and extraction of the classification features. In case of a binary classification, the features are represented by those pattern peaks, which are more abundant in the majority of the samples of one class. These peaks are defined as the characteristic peaks. Thus, two lists of characteristic peaks were generated for each classification. To extract the classification features, the selection of best individual features was applied and the “nearest mean classifier” was used for the classification of test samples and the sample projection onto classification patterns.

## 3. Results

**Figure 4:**
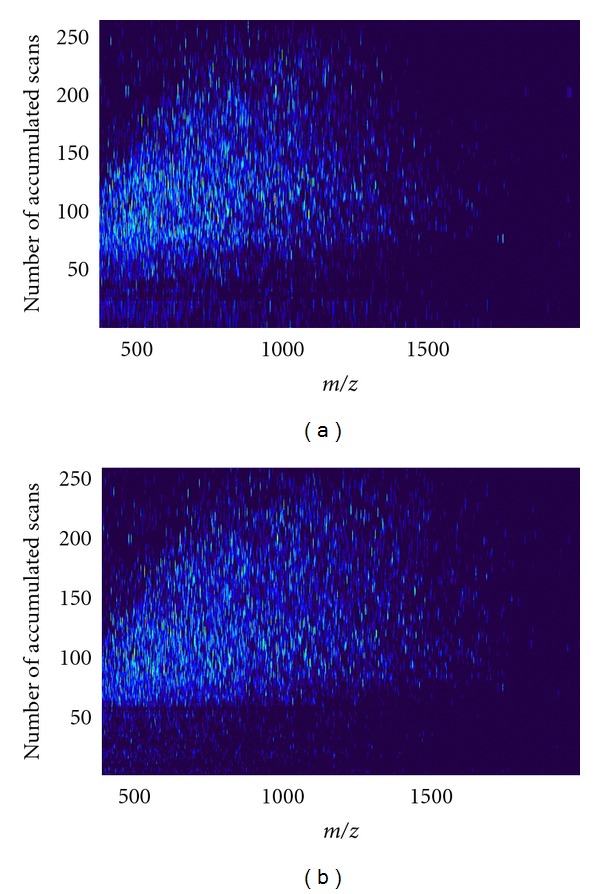
Examples of data (m/z versus number of accumulated scans (corresponds to LC retention time), 10 seconds each) obtained from HPLC-FT-ICR mass spectrometry of aortic (a) and coronary (b) samples. The light spots in the diagrams correspond to individual mass spectral peaks. In the original diagrams the peak intensities are color coded for easy recognition. (Figure modified from reference [9] with kind permission from the publisher).

Examples of two-dimensional data obtained from HPLC-FT-ICR mass spectrometry are shown in Figure 4 for an aortic (a) and a coronary (b) sample.

### 3.1. Comparison and Classification

**Figure 5:**
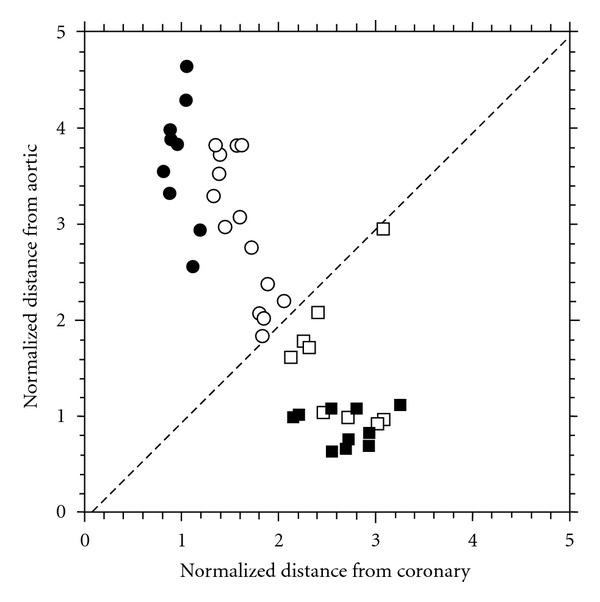
Pattern for the classification of coronary (circular symbols) versus AVD disease (square symbols) samples. Each point represent an individual sample where filled symbols (● and ■) represent supervised classified training samples while open symbols (○ and □) represent unsupervised classified test samples. All samples with exception of two were unambiguously correctly classified. (Figure modified from reference [9] with kind permission from the publisher).

As described in detail in our recent publication [9], the classification using 240 characteristic peaks in the CHD group and 90 characteristic peaks in the AVD group analyzed using LC-FT-ICR MS (Figure 2) resulted in a clear difference in distribution patterns. In Figure 5, the symbols represent both cardiac disorders accumulated on either side of the diagonal line with increased distance from the diagonal related to the specification for the particular type of disease. Above the diagonal, samples from myocardial tissue with CHD and below the diagonal samples from myocardial tissue with AVD are located. Each filled symbol represents an individual supervised classified training sample while each open symbol shows an individual unsupervised classified test sample. The algorithm led to two ambiguous classifications of the unknown test samples. When the mass chromatograms for these ambiguous samples were manually inspected, large gaps in the total ion chromatograms due to unstable electrospray and sudden changes in elution rates could be observed, resulting in difficulties for the algorithm to correctly calibrate and align the datasets. There were intense and stable representative peaks found in both classes. Having a clear difference in proteomic pattern between both myocardial disorders, no interference between aortic and coronary samples was registered, even if the distribution of open spots on either sides of the diagonal (unsupervised classified test samples) displayed a closer distance to the diagonal. The AVD samples showed a closer accumulation pattern of the spots, indicating a more specific classification of the proteins compared to the coronary samples.

### 3.2. Protein Identification Results

Using the digested proteins from chosen samples from patients with CHD as well as with AVD, selected classifier masses were fragmented by collision-induced dissociation in the hexapole collision chamber of the FT-ICR system. Fragmentation spectra of these peptides are used for the identification of the proteins by database search (MASCOT search, [17]). Tables 1(a) and 1(b) show the results obtained from HPLC-FT-ICR MS/MS experiments for CHD and AVD samples, respectively. In samples from patients with CHD, four major proteins were identified from classifiers with a relatively high MASCOT score these are myosin heavy chain alpha isoform, myosin heavy chain beta isoform, adenylate kinase, and troponin T (Table 1(a)). Aortic smooth muscle actin was also identified, with a relatively low MASCOT score, however, still in the useful range when MS/MS of single masses are performed. A larger number of proteins were identified from classifiers in samples from patients with AVD: these were mainly troponin T, desmin, myoglobin, alpha cardiac actin, collagen alpha chain precursor, lumican precursor, tropomyosin I alpha chain, alpha crystallin beta chain, cytochrome C oxydase, ADP/ATP translocase, and serum albumin precursor (Table 1(b)).

Tables 2(a) and 2(b) reveal the protein identification results from database searches with HPLC-MALDI-TOF/TOF spectra of samples from CHD and AVD groups, respectively. In samples from patients with CHD, seven main proteins are found: these were myosin heavy chain beta isoform, alpha crystallin B chain, collagen, elongation factor, hypothetical protein DKFZp686P07163, adenylate kinase, and myosin light chain 2a (Table 2(a)). Like in the FT-ICR results, samples from patients with AVD revealed a larger number of proteins: these were desmin, alpha cardiac actin, myosin heavy chain alpha isoform, myosin heavy chain beta isoform, myosin light chain 2a, ADP/ATP carrier protein, cytochrome C oxydase, alpha-1 (III) collagen, alpha integrin, and an unnamed protein product (Table 2(b)).

**Figure 6:**
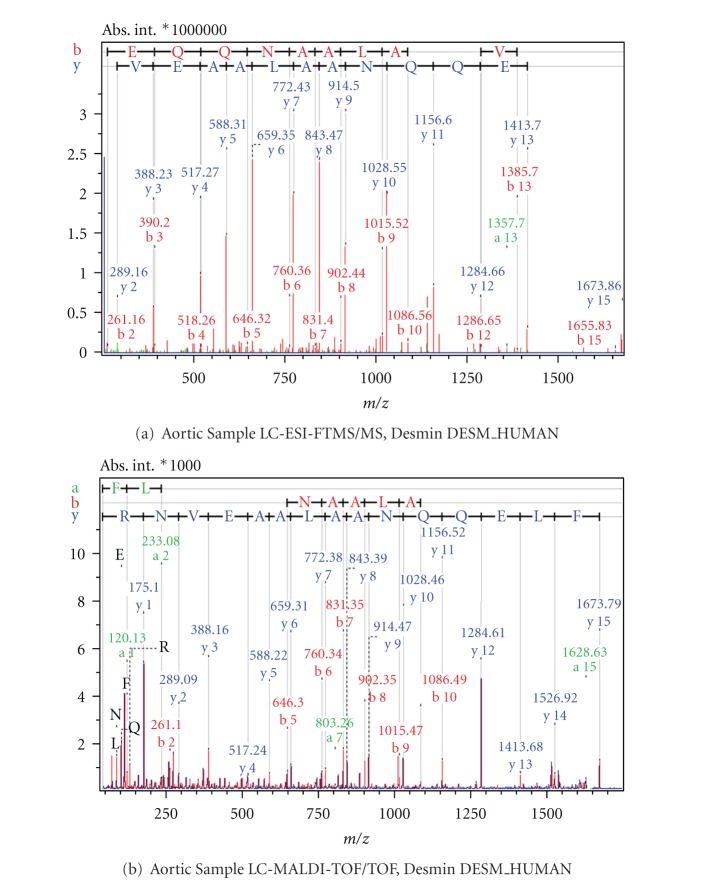
MS/MS spectra of the classifier peptide FLEQQNAALAAEVNR from the sample of an aortic patient. The protein is identified as Desmin upon database search. Spectrum (a) is obtained by LC-ESI-FTMS/MS, spectrum (b) by LC-MALDI-TOF/TOF.

**Figure 7:**
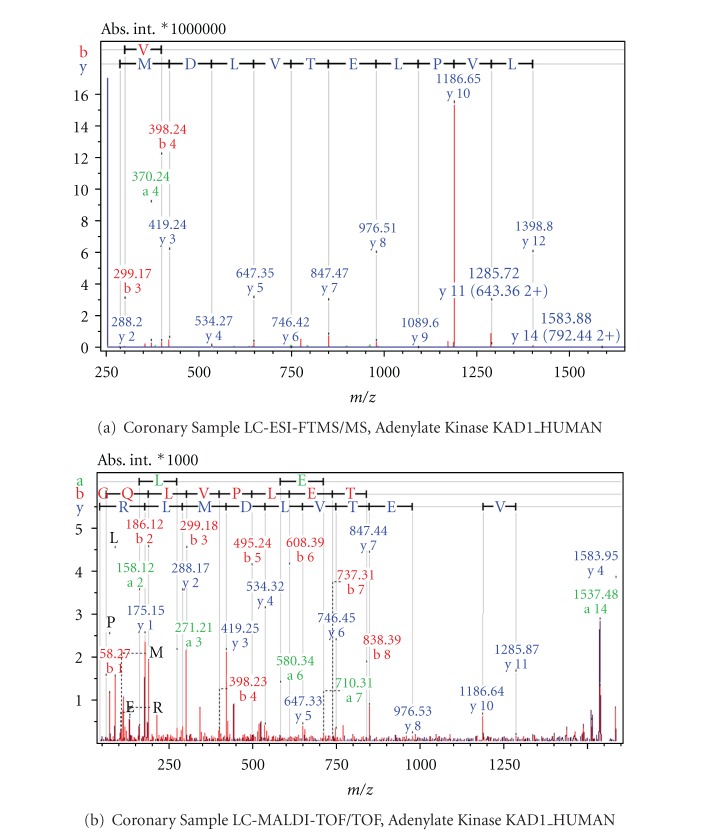
MS/MS spectra of the classifier peptide GQLVPLETVLDMLR from the sample of an aortic patient. The protein is identified as Adenylate Kinase upon database search. Spectrum (a) is obtained by LC-ESI-FTMS/MS, spectrum (b) by LC-MALDI-TOF/TOF.

**Figure 8:**
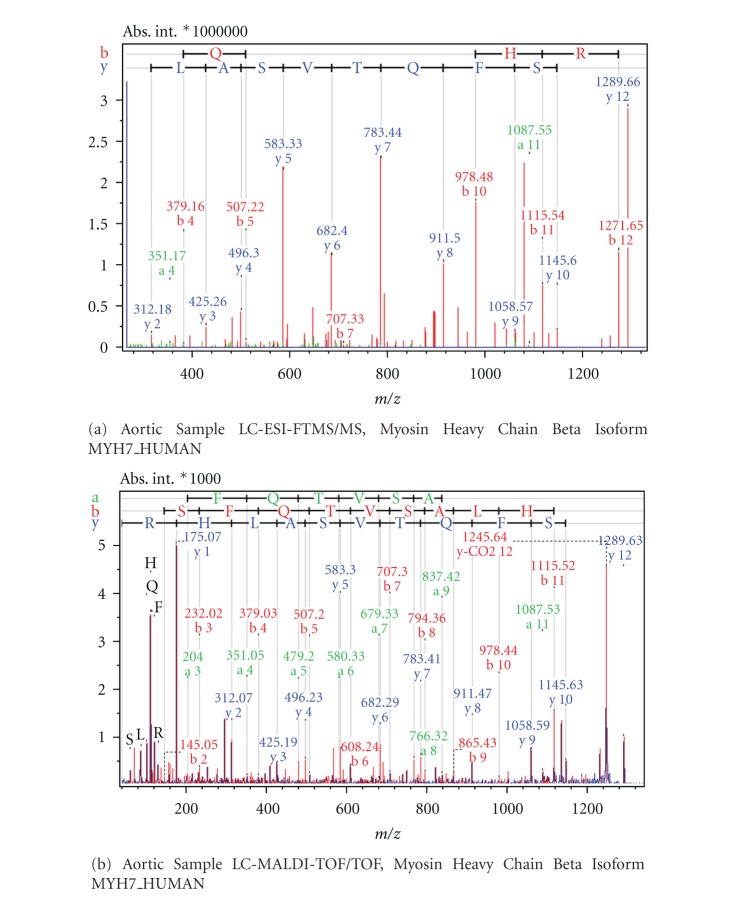
MS/MS spectra of the classifier peptide GSSFQTVSALHR from the sample of an aortic patient. The protein is identified as Myosin Heavy Chain Beta Isoform upon database search. Spectrum (a) is obtained by LC-ESI-FTMS/MS, spectrum (b) by LC-MALDI-TOF/TOF.

**Figure 9:**
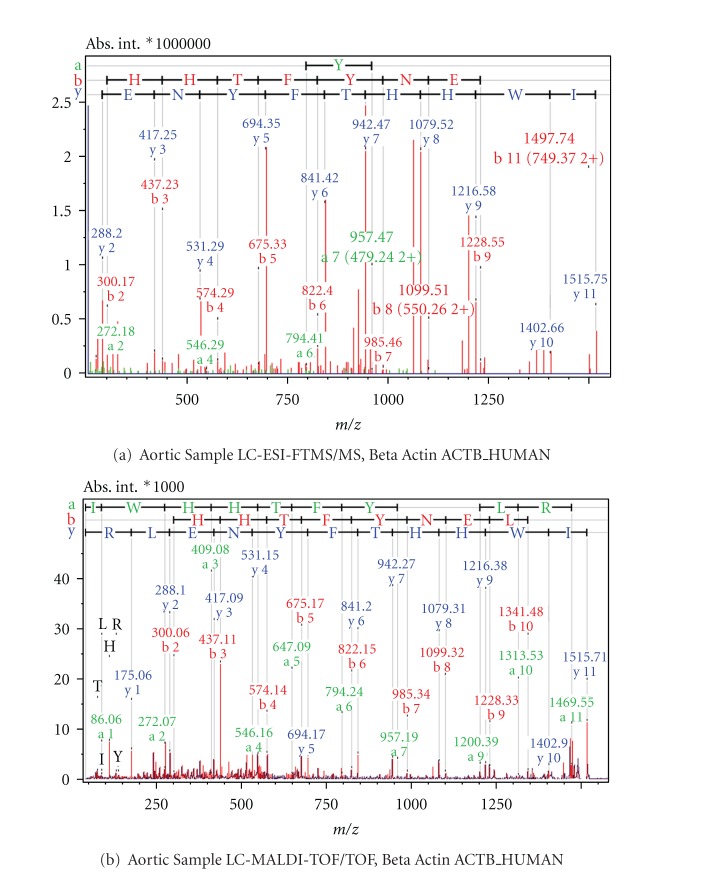
MS/MS spectra of the classifier peptide IWHHTFYNELR from the sample of an aortic patient. The protein is identified as Beta Actin upon database search. Spectrum (a) is obtained by LC-ESI-FTMS/MS, spectrum (b) by LC-MALDI-TOF/TOF.

Examples from MS/MS results acquired with FT-ICR and MALDI-TOF mass spectrometry as comparative cases are shown in Figures 6 to 9.  Figure 6(a) shows the Electrospray Ionisation-FT-ICR MS/MS spectrum obtained from one of the AVD samples leading to the identification of the protein Desmin (DESM_HUMAN), while Figure 6(b) shows the MS/MS spectrum from this sample by MALDI-TOF/TOF mass spectrometry. In both cases, sufficient sequence information is obtained to identify Human Desmin. In the ESI-FT-ICR MS/MS spectrum, the intensities of the y fragment peaks are higher than the b fragments. Yet, the sequence information from both y and b fragments were usable. Similarly, Figures 7(a) and 7(b) show the ESI-FT-ICR MS/MS spectra, and MALDI-TOF/TOF spectra, respectively, obtained from a CHD sample. Adenylate kinase (KAD1_HUMAN) could be identified from any of these two spectra by database search. Here, the ESI-FT-ICR MS/MS spectrum mainly shows y fragments, while in the MALDI-TOF/TOF spectrum y, b, and a fragments are visible. Figures 8(a) and 8(b) show the ESI-FT-ICR MS/MS and MALDI-TOF/TOF mass spectra from an AVD sample, respectively, from any of which the protein myosin heavy chain beta isoform MY7_HUMAN could be identified due to the sequence information. Again here, the ESI-FT-ICR MS/MS spectrum shows mainly the y fragments while MALDI-TOF/TOF spectrum shows b, y, and a fragmentation.  Figure 9 is the identification of beta actin ACTB_HUMAN with ESI-FT-ICR MS/MS (a) and MALDI TOF/TOF (b) again from an AVD sample.

## 4. Discussion

**Figure 10:**
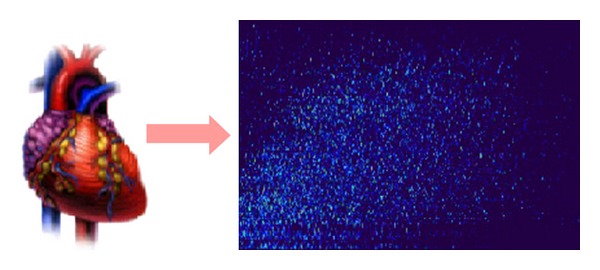
Proteomic profiles of myocardial tissue in two different etiologies of heart failure were investigated using right atrial appendages samples representative for the aortic valve disease and coronary heart disease using a quadrupole/hexapole FT-ICR MS that allowed collision induced dissociation (CID) of selected classifier masses. For comparison and further validation, classifier masses were also fragmented and analyzed using HPLC/Matrix assisted laser desorption ionization (MALDI) time-of-flight/time-of-flight (TOF/TOF) mass spectrometry.

In this work, the sample collection was made in such a way that all patients with CHD selected for this study were free from AVD, and all selected AVD patients were free from CHD. All comparisons presented in this work can, therefore, virtually be considered as to be “diseased” versus “healthy” case against each other. Differences in the mass chromatograms are determined as classifier masses. Thus, proteins identified from classifiers by this *differential mass spectrometry* method are biomarkers in the corresponding disease case (Figure 10).

Some of the identified proteins appear both in the list of CHD samples as well as in the list of the AVD samples. As an example, myosin heavy chain alpha and beta isoforms have been found both in CHD and AVD cases (Tables 1 and 2). However, classifier peptides leading to apparently the same protein by database search were different in CHD samples than in AVD samples. In samples from CHD group, myosin heavy chain alpha isoform was found by database search from MS/MS and identification of the two classifier peptides (Table 1(a)). In samples from AVD group, entirely different classifier peptides led to myosin heavy chain alpha by database search (Table 1(b)). These are displayed in a simplified table (Table 3). A targeted analysis of these potential biomarker peptides could thus result in a simplified diagnostic assay for different etiologies in cardiovascular diseases. The explanation of this observation is suggested as follows. The approach in the present study is the analysis of a peptide mixture resulting from a tryptic digestion of the initial protein mixture. A comparison of the LC-MS data leads to classifier masses for samples with AVD indicating that these particular masses (peptides) are here significantly more abundant than in the samples with CHD, and vice versa. However, since these peptides are the result of a tryptic digestion of the initial proteins, any factor influencing the digestion by trypsin can very well suppress the appearance of some peptides in the digest. If, for example, in CHD samples, some of the tryptic cleavages are suppressed, the comparison will show a higher abundance of these particular digestion products in the aortic samples that they become classifiers for AVD. Similarly, if certain tryptic cleavages are suppressed in samples from AVD patients, the resulting peptides appear more abundant in samples from CHD group, and the comparison will indicate them as classifiers for CHD samples. One of the major factors altering the digestion specificity of trypsin is a posttranslational modification near the cleavage site of the protein. Thus, a tryptic cleavage at this particular site can sometimes be not successful. Heavy groups like large glycans may hinder a cleavage, or even if a cleavage occurs, digested peptide with the modification is too heavy and may be off the detected mass range. It is known that glycosylation can even completely block the tryptic digestion of a protein [22].

Based on the thoughts above, a possible explanation for the different classifier peptides in CHD and AVDs leading to the same myosin may be differences in tryptic digestion patterns caused by posttranslational modifications at different positions. An investigation of the correlation to these differences is the topic of our ongoing work. For this particular study, fragmentations by electron capture dissociation (ECD) or electron transfer dissociation (ETD) are more suitable than collision-induced dissociation (CID), since ECD and ETD protect posttranslational modifications while breaking the peptide backbone bonds.

Another possible reason for altered digestion specificity of trypsin could be the folding geometry. If the protein is not completely unfolded during the digestion process, the tryptic cleavage at particular sites can be sterically hindered. This would then lead to missed cleavages.

A number of proteins in ESI-FT-ICR MS/MS were also identified with the method MALDI-TOF/TOF mass spectrometry with the same peptide sequences. In samples from the CHD group, commonly identified proteins were adenylate kinase, myosin heavy chain beta isoform. In samples from the AVD group, the proteins commonly identified in FT-ICR MS/MS and TOF/TOF were desmin, alpha cardiac actin, myosin heavy chain alpha isoform, myosin heavy chain beta isoform, and ADP/ATP translocase [23].

As described previously, FT-ICR and MALDI-TOF mass spectrometry used different methods of ionization. For the FT-ICR MS, ions were separated by liquid-chromatography and online ionized by electrospray ionization and transferred into mass spectrometer—either directly or after collision-induced dissociation—for detection. The MALDI-TOF mass spectrometric method uses samples in deposited solid phase. Thus, the components in the samples were LC-separated first and fractions were deposited on a MALDI sample plate precoated with a alpha-cyano-4-hydroxy cinnamic acid as a matrix (Pre Spotted Anchor Chip target. Bruker Daltonik, Bremen, Germany). This plate was inserted into the ion source of the TOF mass spectrometer, irradiated with the laser beam, and the ions were generated by matrix assisted laser desorption/ionization and detected. These ions are in general singly charged. Although compounds in both MALDI and electrospray ionization become mildly ionized, due to the different ionization techniques in MALDI-TOF and FT-ICR instruments, and to the non-online method in the LC-MALDI-TOF, some of the ions may have different abundances than in the on-line system. Thus, some of the FT-ICR MS/MS or MALDI-TOF/TOF dissociations of classifiers could not be performed as the abundance in the corresponding system was low. One of the examples is the alpha crystallin B chain [24–27] in the CHD samples which is intensively found in the MALDI-TOF spectra, fragmented (TOF/TOF), detected, and identified, which was not sufficiently abundant in the experiments with the FT-ICR MS. Thus, no results regarding detection of alpha crystallin B chain have been displayed in the CHD samples by FT-ICR MS.

As an overview, the bottom-up proteomic approach was applied to proteins in human cardiac muscle tissue samples from two groups of patients in this study. The selection of patients enabled the examination of two clearly separated case etiologies. Proteins in the tissue samples were digested by trypsin, and the digest containing a mixture of peptides was analyzed by mass spectrometry after liquid chromatographic separation. The use of high-resolution mass spectrometry (in this case FT-ICR MS) allowed to resolve and display the mass spectrometric peaks in this complex picture of the LC-MS results and to compare both separate disease forms. By comparison of LC-MS diagrams, the classifier masses could be clearly identified. These were selected in the subsequent experiments and fragmented (LC-FT-ICR MS/MS), in order to identify the proteins which had generated the classifier masses. The comparison clearly separated both disease forms while the analysis and identification of the proteins which led to the classifiers helped to study the biomarkers related to CHD and AVD. An additional work for comparing LC-MALDI-TOF mass spectrometry and LC-MALDI TOF/TOF for the MS/MS fragmentation has also be performed.

The unique patient selection in this study, combined with the bottom-up proteomic approach using liquid chromatography and high resolution mass spectrometry, seems to be highly efficient in determination of the differences between selected disease groups. The *differential high-resolution mass spectrometry* performed subsequently to characterize the related proteins did require the MS/MS of the classifier masses only.

This study of the heart muscle tissue samples helped establish a first picture of the proteomic appearance of two virtually independent etiologies of heart disease. As our main target is to diagnose cardiac disease less invasively and directly, we are currently investigating blood plasma samples from CHD and AVD patients in order to identify the differences with the same technique using differential high-resolution mass spectrometry.

## Figures and Tables

**Table:** (a) FT-ICR MS/MS database search identification of classifier proteins from CHD patients

Protein ID in coronary samples	Peptide sequence	Sequence tag	*m/z* (charge state)	Mascot score
(P13533) Myosin heavy chain, cardiac muscle alpha isoform (MyHC-alpha) MYH6_HUMAN	KLAEKDEEMEQAK	1577–1589	774.884 (2+), 516.925 (3+)	65 (92), 28
	NLQEEISDLTEQLGEGGKNVHELEKVR	1506–1532	766.896 (4+)	113

(P12883) Myosin heavy chain, cardiac muscle beta isoform (MyHC-beta) MYH7_HUMAN	KLAEKDEEMEQAK	1575–1587	774.884 (2+), 516.925 (3+)	65, 28

(P00568) Adenylate kinase isoenzyme 1 (EC 2.7.4.3) (ATP-AMP transphosphorylase) (AK1) (Myokinase) KAD1_HUMAN	GQLVPLETVLDMLR	64–77	792.447 (2+)	105

(P45379) Troponin T, cardiac muscle (TnTc) (Cardiac muscle troponin T) (cTnT) TNNT2_HUMAN	VLAIDHLNEDQLR	227–239	768.415 (2+)	98 (86)

(P62736) Actin, aortic smooth muscle (Alpha-actin-2) ACTA_HUMAN	MQKEITALAPSTMK	315–328	774.912 (2+)	18

**Table:** (b) FT-ICR MS/MS database search identification of classifier proteins in AVD patients

Protein ID in aortic samples	Peptide sequence	Sequence tag	*m/z* (charge state)	Mascot score
(P45379) Troponin T, cardiac muscle (TnTc) (Cardiac muscle troponin T) (cTnT) TNNT2_HUMAN	DLNELQALIEAHFENR	107–122	956.482 (2+)	93

(P17661) Desmin DESM_HUMAN	FLEQQNAALAAEVNR	127–141	837.426 (2+)	90

(P02144) Myoglobin MYG_HUMAN	HPGDFGADAQGAMNK	119–133	505.894 (3+)	71 (20)

(P60709) Actin, cytoplasmic 1 (Beta-actin) ACTB_HUMAN or	IWHHTFYNELR	85–95	505.922 (3+)	70 (53) (55)
(P68032) Actin, alpha cardiac (Alpha-cardiac actin) ACTC_HUMAN	IWHHTFYNELR	87–97	758.379 (2+), 505.922 (3+)	53, 61

(P51884) Lumican precursor (Keratan sulfate proteoglycan lumican) (KSPG lumican) LUM_HUMAN	ILGPLSYSK LKEDAVSAAFK	297–305 171–181	489.288 (2+) 589.825 (2+)	29 60

(P12111) Collagen alpha-3 (VI) chain precursor CO6A3_HUMAN	VAVVQYSDR	1067–1075	518.776 (2+)	59

(P12883) Myosin heavy chain, cardiac muscle beta isoform (MyHC-beta) MYH7_HUMAN	RKLEGDLK (also in MYH6_Human)	1053–1060	479.788 (2+)	21
	AQLEFNQIK	1561–1569	545.799 (2+)	25
	GSSFQTVSALHR	641–652	645.334 (2+)	25 (35) (62)
(P13533) Myosin heavy chain, cardiac muscle alpha isoform (MyHC-alpha) MYH6_HUMAN	RKLEGDLK	1055–1062	479.788 (2+)	21
	AQLEFNQIK	1563–1571	545.799 (2+)	25 (26)
	GSSFQTVSALHR	643–654	645.334 (2+), 430.559 (3+)	25 (17), 47
	GKLSYTQQMEDLKR	1306–1319	566.295 (3+)	37

(P09493) Tropomyosin 1 alpha chain (Alpha-tropomyosin) TPM1_HUMAN	MEIQEIQLK	141–149	566.309 (2+)	36

(P02768) Serum albumin precursor ALBU_HUMAN	KYLYEIAR	161–168	528.299 (2+)	29

(P02511) Alpha crystallin B chain (Alpha(B)-crystallin) (Rosenthal fiber component) (Heat-shock) pro-CRYAB_HUMAN	HFSPEELK	83–90	493.751 (2+)	28

(P09669) Cytochrome c oxidase polypeptide VIc precursor (EC 1.9.3.1) COX6C_HUMAN	KAGIFQSVK	67–75	489.293 (2+)	28

(P12235) ADP/ATP translocase 1 (Adenine nucleotide translocator 1) (ANT 1) ADP, ATP carrier protein (ADT1_HUMAN)	TAVAPIER	23–30	856.487 (1+)	24 (22)

(P35555) Fibrillin-1 precursor FBN1_HUMAN	TICIETIK	843–850	489.271 (2+)	23

(P02768) Serum albumin precursor ALBU_HUMAN	KYLYEIAR	161–168	528.298 (2+)	22

(P19429) Troponin I, cardiac muscle (Cardiac troponin I) TNNI3_HUMAN	AKESLDLR	162–169	466.264 (2+)	19
(P06576) ATP synthase beta chain, mitochondrial precursor (EC 3.6.3.14) ATPB_HUMAN	FLSQPFQVAEVFTGHMGK	463–480	675.008 (3+)	16

**Table:** (a) HPLC/MALDI TOF/TOF database search identification of classifier proteins in CHD patients

Protein ID of coronary samples	Peptide sequence	Sequence tag	*m/z* (charge state)	Mascot score	Protein summary score
Collagen HSCOLL NID: -Homo sapiens CAA23761 or AF004877 NID: -Homo sapiens AAB93981	GYPGNIGPVGAAGAPGPHGPVGPAGK 3: Hydroxyl(P) 15: Hydroxyl(P) GYPGNIGPVGAAGAPGPHGPVGPAGK 3: Hydroxyl(P) 15: Hydroxyl(P)	327–352 949–974	2284.147	185 (91) 91	88

(P12883) Myosin heavy chain, cardiac muscle beta isoform (MyHC-beta) MYH7_HUMAN (Displayed; Variant CMH1-VAR_019864)	VIQYFAVIAAIGDR	191–204	1535.858.	72	88 (41.20)

(Q05639) Elongation factor 1-alpha 2 (EF-1-alpha-2) (Elongation factor 1 A-2) (eEF1A-2) (Statin S1) EF1A2_HUMAN	VETGILRPGMVVTFAPVNITTEVK	267–280	2571.421	55	74.50

Hypothetical protein DKFZp686P07163. -Homo sapiens (Human). Q5HYB7_HUMAN	SSSLLIPPLETALANFSSGPEGGVMQPVR	19–47	2954.529	80 (66)	105 (94.90)

AX885183 NID: -Homo sapiens CAE99297 or AX885189 NID: -Homo sapiens CAE99303 or AX885185 NID: -Homo sapiens CAE99299 or alpha-crystallin chain B (validated) -human CYHUAB or AF007162 NID: -Homo sapiens AAC19161 or AX888028 NID: -Homo sapiens CAE93953	LFDQFFGEHLLESDLFPTSTSLSPFYLRPPSFLR LFDQFFGEHLLESDXFPTSTSLSPFYLRPPSFLR	23–56 18–51 98–131 23–56 23–56	4004.027	106 (34)	133 (56.90)

(P02511) Alpha crystallin B chain (Alpha(B)-crystallin) (Rosenthal fiber component) (Heat-shock) pro CRYAB_HUMAN	LFDQFFGEHLLESDLFPTSTSL	23–44	2543.234	128	131

Crystallin, alpha B (Homo sapiens) gi∣13937813	LFDQFFGEHLLESDLFPTSTSL	23–44	2543.234	110	111

Crystallin, alpha B (Homo sapiens) gi∣4503057	LFDQFFGEHLLESDLFPTSTSL	23–44	2543.234	95	95.3

Adenylate kinase (EC 2.7.4.3) 1-human (tentative sequence) KIHUA or AK1 protein (Adenylate kinase 1). -Homo sapiens, Q6FGX9_HUMAN or BC001116 NID: Homo sapiens AAH01116 or Adenylate kinase 1. -Homo sapiens (Human). Q5T9B7_HUMAN	GQLVPLETVLDMLR	64–77 64–77 64–77 79–93	1583.882	64 (49)	86.10 (69.80)
(MLRA_HUMAN) Myosin regulatory light chain 2, atrial isoform (Myosin light chain 2a) (MLC-2a) (MLC2a) (Myosin regulatory light chain 7) Myosin regulatory light Q01449	QLLLTQADKFSPAEVEQMFALTPMDLAGNIDYK	129–161	3697.849	106	131
(P45379) Troponin T, cardiac muscle (TnTc) (Cardiac muscle troponin T) (cTnT) TNNT2_HUMAN	VLAIDHLNEDQLR	227–239	1535.81	58	85

**Table:** (b) HPLC/MALDI TOF/TOF database search identification of classifier proteins in AVD patients

Protein ID of aortic samples	Peptide sequence	Sequence tag	*m/z*	Mascot score	Protein summary score
(P17661) Desmin DESM_HUMAN	FLEQQNAALAAEVNR	127–141	1673.860	115	133.00

mutant desmin (Homo sapiens) gi∣21358854	FLEQQNAALAAEVNR	128–142	1673.860	99 (65)	117 (84.30)

(P60709) Actin, cytoplasmic 1 (Beta-actin) ACTB_HUMAN or	IWHHTFYNELR	85–95	1515.749	82	100
(P63261) Actin, cytoplasmic 2 (Gamma-actin) ACTG_HUMAN or	IWHHTFYNELR	85–95			
(P68133) Actin, alpha skeletal muscle (Alpha-actin 1) ACTS_HUMAN or	IWHHTFYNELR	85–95			
(P68032) Actin, alpha cardiac (Alpha-cardiac actin) ACTC_HUMAN	IWHHTFYNELR	87–97			

(P12883) Myosin heavy chain, cardiac muscle beta isoform (MyHC-beta) MYH7_HUMAN or	GSSFQTVSALHR	641–652	1289.660	79	87.30
(P13533) Myosin heavy chain, cardiac muscle alpha isoform (MyHC-alpha) MYH6_HUMAN	GSSFQTVSALHR	643–654	1289.660	79	85.70

MSTP161 (Homo sapiens) gi∣33338222	SFPNLAFIR	108–116	1064.589	74	97.40

Myosin light chain 2a (Homo sapiens) gi∣10864037	SLCYIITHGDEKEE 3: Carbamidomethyl (C)	162–175	1693.774	66	122

actin-like protein (Homo sapiens) gi∣62421180	IWHHTFYNELR	2–12	1515.749	64	88.40

Myosin heavy chain alpha subunit gi∣386971	AQLEFNQIK	13–21	1090.589	60	81

alpha-1 type III collagen gi∣180413 or	GDKGETGER	7–15	948.438	57	87
unnamed protein product (Homo sapiens) gi∣1340174 or		28–36			80.1
alpha1 (III) collagen (Homo sapiens) gi∣30054 or		141–153			74.50
alpha-1 (III) collagen (Homo sapiens) gi∣930045 or		945–953			71.40
III preprocollagen alpha 1 chain (Homo sapiens) gi∣16197601		1092–1100			70.10

(P12235) ADP, ATP carrier protein, heart/skeletal muscle isoform T1 (ADP/ATP translocase 1) ADT1_HUMAN or	TAVAPIER	23–30	856.489	49	65.10
(P05141) ADP, ATP carrier protein, fibroblast isoform (ADP/ATP translocase 2) ADT2_HUMAN or	TAVAPIER	23–30	856.489	49	65.10
(P12236) ADP, ATP carrier protein, liver isoform T2 (ADP/ATP translocase 3) ADT3_HUMAN	TAVAPIER	23–30	856.489	49	65.10

Cytochrome c oxidase subunit Va, (COX5A protein). -Homo sapiens (Human). Q8TB65_HUMAN	RLNDFASTVR	98–107	1178.628	49	70.40
(P12883) Myosin heavy chain, cardiac muscle beta isoform (MyHC-beta) MYH7_HUMAN or	ILYGDFR	713–719	883.467	46	56.20
(P13533) Myosin heavy chain, cardiac muscle alpha isoform (MyHC-alpha) MYH6_HUMAN	ILYGDFR	715–721	883.467	46	56.20

(P12883) Myosin heavy chain, cardiac muscle beta isoform (MyHC-beta) MYH7_HUMAN or (P13533) Myosin heavy chain, cardiac muscle alpha isoform (MyHC-alpha) MYH6_HUMAN	AVVEQTER	1689–1697 1692–1699	931.484	38	49.20

unnamed protein product (Homo sapiens) gi∣34533821	FLLVGQTMSTLLDEDLTK	495–512	2024.062	29	47.90

Myosin, heavy polypeptide 7, cardiac muscle, beta variant (Homo sapiens) gi∣62088996 or cardiac beta myosin heavy chain (Homo sapiens) gi∣29727	AGLLGLLEEMRDER 10: Oxidation (M)	406–419	1617.826	29	42

(O14958) Calsequestrin, cardiac muscle isoform precursor (Calsequestrin 2) CASQ2_HUMAN	EHQRPTLR	243–250	1036.565	24	41.60

alpha integrin interacting protein 63 (Homo sapiens) gi∣4468915	ESVSSFVR	27–35	910.463	25	39.60

Myosin, heavy polypeptide 7, cardiac muscle, beta variant (Homo sapiens) gi∣62088996	GSSFQTVSALHR	271–291	1289.660	47	63.60

**Table 3:** Classifier peptides in aortic and coronary diseases identifying myosin heavy chain alpha and beta isoforms as potential biomarkers.

	Classifier peptides	
Identified protein	CHD disease	AVD disease
Myosin heavy chain alpha isoform	KLAEKDEEMEQAK,	RKLEGDLK,
	NLQEEISDLTEQLGEGGKNVHELEKVR	AQLEFNQIK,
		GSSFQTVSALHR,
		GKLSYTQQMEDLKR

Myosin heavy chain beta isoform	KLAEKDEEMEQAK	RKLEGDLK,
		AQLEFNQIK,
		GSSFQTVSALHR

## References

[1] Hansson GK (2005). Mechanisms of disease: inflammation, atherosclerosis, and coronary artery disease. *New England Journal of Medicine*.

[2] Maron BJ, Ferrans VJ, Roberts WC (1975). Myocardial ultrastructure in patients with chronic aortic valve disease. *American Journal of Cardiology*.

[3] De La Cuesta F, Alvarez-Llamas G, Gil-Dones F (2009). Tissue proteomics in atherosclerosis: elucidating the molecular mechanisms of cardiovascular diseases. *Expert Review of Proteomics*.

[4] De La Cuesta F, Alvarez-Llamas G, Maroto AS (2011). A proteomic focus on the alterations occurring at the human atherosclerotic coronary intima. *Molecular and Cellular Proteomics*.

[5] Zhang J, Guy MJ, Norman HS (2011). Top-down quantitative proteomics identified phosphorylation of cardiac troponin I as a candidate biomarker for chronic heart failure. *Journal of Proteome Research*.

[6] Gerszten RE, Asnani A, Carr SA (2011). Status and prospects for discovery and verification of new biomarkers of cardiovascular disease by proteomics. *Circulation Research*.

[7] Huang WJ, Zhou R, Zeng XR (2011). Comparative proteomic analysis of atrial appendages from rheumatic heart disease patients with sinus rhythm and atrial fibrillation. *Molecular Medicine Reports*.

[8] Dubois E, Fertin M, Burdese J, Amouyel P, Bauters C, Pinet F (2011). Cardiovascular proteomics: translational studies to develop novel biomarkers in heart failure and left ventricular remodeling. *Proteomics*.

[9] Baykut D, Grapow M, Bergquist M (2006). Molecular differentiation of ischemic and valvular heart disease by liquid chromatography/fourier transform ion cyclotron resonance mass spectrometry. *European Journal of Medical Research*.

[10] Ramström M, Ivonin I, Johansson A (2004). Cerebrospinal fluid protein patterns in neurodegenerative disease revealed by liquid chromatography-Fourier transform ion cyclotron resonance mass spectrometry. *Proteomics*.

[11] Bergquist J, Palmblad M, Wetterhall M, Håkansson P, Markides KE (2002). Peptide mapping of proteins in human body fluids using electrospray ionization fourier transform ion cyclotron resonance mass spectrometry. *Mass Spectrometry Reviews*.

[12] Wu SL, Choudhary G, Ramström M, Bergquist J, Hancock WS (2003). Evaluation of shotgun sequencing for proteomic analysis of human plasma using HPLC coupled with either ion trap or Fourier transform mass spectrometry. *Journal of Proteome Research*.

[13] Bergquist J (2003). FTICR mass spectrometry in proteomics. *Current Opinion in Molecular Therapeutics*.

[14] Ekegren T, Hanrieder J, Aquilonius SM, Bergquist J (2006). Focused proteomics in post-mortem human spinal cord. *Journal of Proteome Research*.

[15] Caravatti P, Allemann M (1991). ‘The infinity cell‘: a new trapped-ion cell with radiofrequency covered trapping electrodes for Fourier transform ion cyclotron resonance mass spectrometry. *Organic Mass Spectrometry*.

[16] Pappin DJC, Rahman D, Hansen HF, Bartlet-Jones M, Jeffery W, Bleasby AJ, Burlingame AL, Carr SA (1996). Chemistry Mass Spectrometry and Peptide-Mass Databases: evolution of methods for the rapid identification and mapping of cellular proteins. *Mass Spectrometry in the Biological Sciences*.

[17] MASCOT http://www.matrixscience.com/.

[18] Pappin DJC, Hojrup P, Bleasby AJ (1993). Rapid identification of proteins by peptide-mass fingerprinting. *Current Biology*.

[19] Shi SDH, Drader JJ, Freitas MA, Hendrickson CL, Marshall AG (2000). Comparison and interconversion of the two most common frequency-to-mass calibration functions for Fourier transform ion cyclotron resonance mass spectrometry. *International Journal of Mass Spectrometry*.

[20] Suckau D, Resemann A, Schuerenberg M, Hufnagel P, Franzen J, Holle A (2003). A novel MALDI LIFT-TOF/TOF mass spectrometer for proteomics. *Analytical and Bioanalytical Chemistry*.

[21] Holle A, Haase A, Kayser M, Höhndorf J (2006). Optimizing UV laser focus profiles for improved MALDI performance. *Journal of Mass Spectrometry*.

[22] Deininger SO, Rajendran L, Lottspeich F (2003). Identification of teleost Thy-1 and association with the microdomain/lipid raft reggie proteins in regenerating CNS axons. *Molecular and Cellular Neuroscience*.

[23] Portman MA (2000). Adenine nucleotide translocator in heart. *Molecular Genetics and Metabolism*.

[24] Wang X, Klevitsky R, Huang W, Glasford J, Li F, Robbins J (2003). *α*B-Crystallin Modulates Protein Aggregation of Abnormal Desmin. *Circulation Research*.

[25] Bhat SP, Horwitz J, Srinivasan A, Ding L (1991). *α*B-crystallin exists as an independent protein in the heart and in the lens. *European Journal of Biochemistry*.

[26] Lutsch G, Vetter R, Offhauss U (1997). Abundance and location of the small heat shock proteins HSP25 and *α*B- crystallin in rat and human heart. *Circulation*.

[27] Horwitz J (1992). *α*-Crystallin can function as a molecular chaperone. *Proceedings of the National Academy of Sciences of the United States of America*.

